# Use of Estonian Biobank data and participant recall to improve Wilson’s disease management

**DOI:** 10.1038/s41431-024-01767-9

**Published:** 2024-12-14

**Authors:** Miriam Nurm, Anu Reigo, Tarmo Annilo, Toomas Toomsoo, Margit Nõukas, Tiit Nikopensius, Vasili Pankratov, Tuuli Reisberg, Georgi Hudjashov, Estonian Biobank Research Team, Georgi Hudjashov, Andres Metspalu, Lili Milani, Tõnu Esko, Reedik Mägi, Mari Nelis, Toomas Haller, Neeme Tõnisson

**Affiliations:** 1 Estonian Genome Center, Institute of Genomics, University of Tartu, Tartu, Estonia; 2 Confido Medical Center, Tartu, Estonia; 3 School of Natural Sciences and Health, Tallinn University, Tallinn, Estonia; 4 Core Facility of Genomics, Institute of Genomics, University of Tartu, Tartu, Estonia; 5 Genetics and Personalized Medicine Clinic, Tartu University Hospital, Tartu, Estonia

**Keywords:** Metabolic disorders, Personalized medicine, Medical genomics

## Abstract

Population-based biobanks enable genomic screening to support initiatives that prevent disease onset or slow its progression and to estimate the prevalence of genetic diseases in the population. Wilson’s disease (WD) is a rare genetic copper-accumulation disorder for which timely intervention is crucial, as treatment is readily available. We studied WD in the Estonian Biobank population to advance patient screening, swift diagnosis, and subsequent treatment. Combined analysis of genotype and phenotype data from electronic health records (EHRs) consolidated at the Estonian biobank led to the identification of 17 individuals at high risk of developing WD, who were recalled for further examination and deep phenotyping. All recall study participants, regardless of phenotype, age, and prior WD diagnosis, had low serum ceruloplasmin and copper levels, and 87% also exhibited signs of early to late neurodegeneration. The p.His1069Gln variant in *ATP7B*, a prevalent pathogenic mutation, showed a striking four- to five-fold enrichment in Estonians compared with other populations. Based on our analysis of genetic and nationwide health registry data, we estimate that WD remains underdiagnosed and undertreated in Estonia. Our study demonstrates that personalized medicine, implemented with the collaboration of medical professionals, has the potential to reduce the healthcare burden by facilitating the accurate diagnosis of rare genetic diseases. To our knowledge, this report is the first to describe a large-scale national biobank–based study of WD.

## Introduction

Wilson’s disease (WD), caused by a defect in the *ATP7B* gene, is a rare autosomal recessive copper metabolism disorder that causes copper accumulation in the body. It typically presents with hepatic or neurological symptoms [1], but it exhibits noteworthy phenotypic heterogeneity and a wide range in the age of onset [2], making its diagnosis challenging. *ATP7B* variant c.3207 C > A (p.His1069Gln) is considered to be the predominant WD-causing variant in Europe [3] and has been proposed to have arisen in Eastern Europe [4].

The genetic prevalence of WD has recently been estimated to be 1:7,000–1:8,000 [5–7] with a carrier frequency of about 1:50 globally [6, 7]. Its estimated clinical prevalence is 1:30,000–1:50,000 and has remained the same since 1984 [8]. The discrepancy between the genetic and clinical prevalence of WD has been attributed to variable penetrance [8], misdiagnosis or underdiagnosis [6, 7], milder phenotypes [5], and other modifying factors [9].

Magnetic resonance imaging (MRI) of the brain is used in routine clinical practice to identify WD-related lesions, most commonly in the lentiform nucleus, caudate nucleus, pons, thalamus, and midbrain; T2-weighted imaging has the greatest sensitivity for its purpose [10]. Transcranial sonography (TCS) is a time- and cost-efficient method that has been used to diagnose various movement disorders [11]. Its sensitivity in the detection of lentiform nucleus hyperechogenicity (LN + ), indicative of WD, has been studied [12, 13].

The main objective of our study was to investigate how biobank data from the Estonian Biobank (EstBB) – genotypic data and electronic health records (EHRs) combined - can be used to provide an overview of WD prevalence in Estonia as well as recommendations for management. We also conducted genotype-first recalls of EstBB participants to demonstrate the practical application of biobank data in detecting previously underdiagnosed rare disease cases.

## Materials and methods

### Study design and cohort overview

**Fig. 1:**
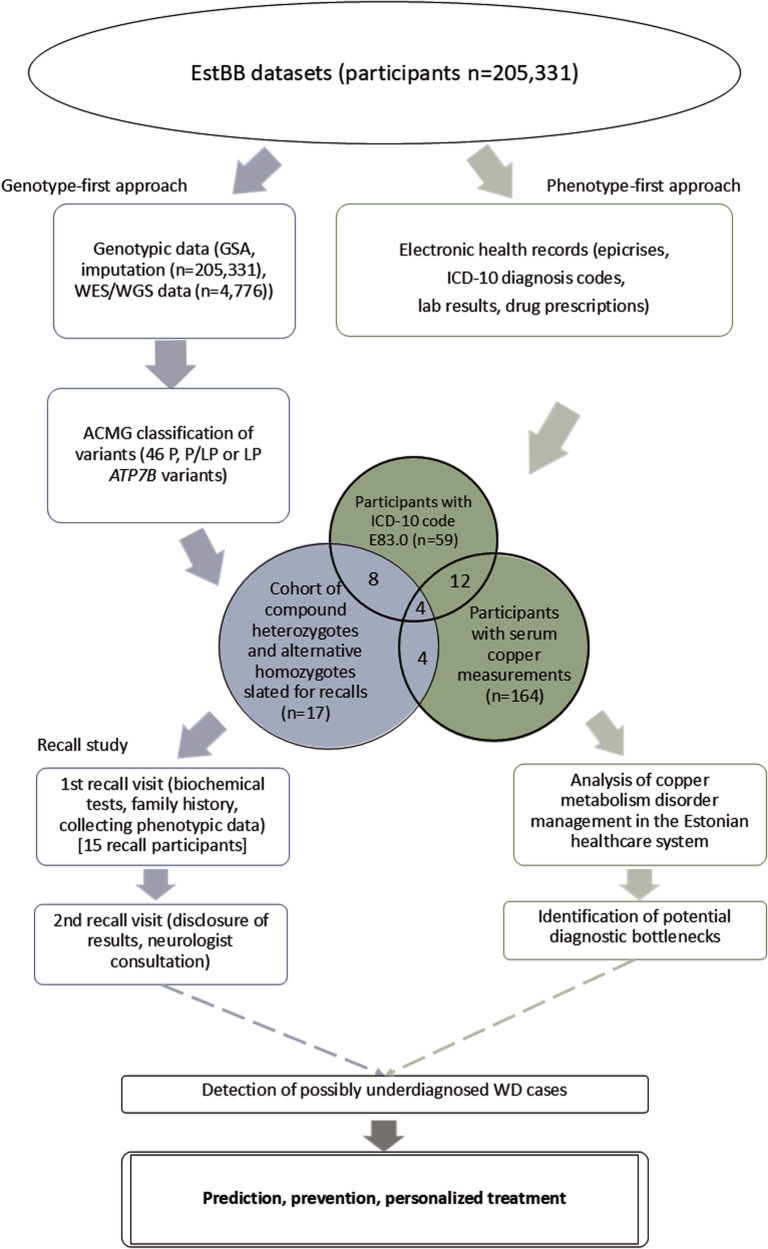
Diagram of study flow. GSA global screening array (Illumina), ICD-10 International Classification of Diseases, 10th edition; ACMG American College of Medical Genetics, *P* pathogenic, LP likely pathogenic, EstBB Estonian Biobank, WD Wilson’s disease, WES whole exome sequencing, WGS whole genome sequencing.

The study employed a combined genotype-phenotype analysis in the EstBB population to investigate WD prevalence and identify potential cases (Fig. 1). The EstBB maintains data, including genotypic and phenotypic data (self-reported medical histories, lifestyle information, EHRs), from 210,000 participants (about 20% of Estonia’s adult population) [14]. Participants provided broad written consent, allowing EstBB to re-contact them and update their data through EHRs and national health registry linkage [15]. As a result, both retro- and anterograde health records are available. This study was conducted using directly genotyped data from Infinium Global Screening Array (v1.0 and 2.0; Illumina Inc., San Diego, CA, USA), enriched by imputed genotype data, available for 205,331 participants, and next-generation sequencing (NGS) data, available for 4776 participants (whole-exome sequencing, *n* = 2356; whole-genome sequencing, *n* = 2420). Technical details on direct and imputed genotyping and annotation at EstBB have been provided previously [16, 17]. This study received approval from the Estonian Committee on Bioethics and Human Research.

### Genotype-first approach

Variants in the *ATP7B* gene and flanking regions (chr13:52499572–52591261, RefSeq build GRCh37) were identified at the EstBB using the high-coverage sequencing, genotyping array, and imputed data. A two-stage filtration process was employed, with consideration of *in-silico* pathogenicity scores (MetaLR [18], CADD [19], and VEP IMPACT [20]), allele frequencies, and regulatory element overlap, and comparison with clinical variant databases (ClinVar [21] and WilsonGen [22]).

Initially, variants with “moderate” and “high” IMPACT categories were chosen (Supplementary Fig. S1). Variants with “modifier” IMPACT designations and known overlap of regulatory elements (enhancer, promotor, or promotor flanking regions from the Ensembl database [23]) were added to this sample. Variants outside the predefined *ATP7B* coordinates and those with allele frequencies >1% were excluded. Variants with “pathogenic” (P) and “likely pathogenic” (LP) ClinVar designations were selected. Those without both CADD scores ≥10 and “deleterious” MetaLR predictions were excluded.

In the second filtration stage, variants meeting any of the following criteria were excluded: null-imputation rows (indicating failed variant carrier detection), minor allele frequency (MAF) > 0.0054, and confirmation of benignity in a published study or “benign” or ”likely benign” designation in the WilsonGen or ClinVar database. The filtration was performed in 2020–2021 with the database versions available at the time. Variant classification followed the American College of Medical Genetics/American Molecular Pathologists (ACMG/AMP) 2015 guidelines [24]. The technical details of the follow-up exome sequencing are available in Supplementary Material S1.

### Investigating management of copper metabolism disorders in the healthcare system

As part of investigating the management of copper metabolism disorders in the Estonian healthcare system, a phenotype-first approach was used to identify EstBB participants presenting as potential WD cases but without a formal WD diagnosis. The EHR data of EstBB participants who were diagnosed at least once with a copper metabolism disorder [International Classification of Diseases (ICD-10) code E83.0] and who had copper concentrations measured in serum or 24-h urine, were analysed. Epicrises, ICD-10 codes, drug prescription data (Anatomical Therapeutic Classification/Defined Daily Dose codes, dosages, and purchases), and laboratory analyses covering up to 18 years (2004–2023) were retrieved for all study participants. Missing analysis units were inferred from epicrises when available. Individuals with E83.0 diagnoses were categorized as unlikely, possible but unlikely, and possible WD candidates. The criteria for exclusion based on insufficient data included the lack of recurrent E83.0 diagnosis or corresponding epicrisis and the lack of symptoms clearly indicative of WD (presented in epicrises or as ICD-10 codes). To be classified as possible WD, at least two of the following four criteria had to be met: presence of Kayser-Fleischer rings, WD treatment history, low serum copper or ceruloplasmin concentration (or no measurement available), and presentation of overlapping hepatological and neurological/psychiatric symptoms indicative of WD [25]. In unclear cases, a history of alcohol abuse along with the presence of liver damage and the age of first symptom onset (>35 years) were considered as arguments for an “unlikely” classification. EstBB EHRs were further mined for studying the indications behind serum copper testing as part of the management of individuals with copper metabolism disorders. Statistics from the Estonian Health Insurance Fund and Estonian Medicines Agency were explored to compare WD and E83.0 prevalence in Estonia.

### Recall study

Genetic findings for EstBB participants identified as alternative homozygotes or compound heterozygotes for P or LP *ATP7B* gene variants were validated using Sanger sequencing with custom primers at the Estonian Genome Center Core Facility [26]. The recall cohort was chosen based on genetic data after EHR consultation. The study was conducted in 2022–2023. All recall cohort participants received invitation letters with study background information that prompted them to schedule initial recall visits. No personal genetic risk information was disclosed in the letters. Non-responders received a follow-up invitation letter 1 month later, and those who still did not respond were contacted by telephone by biobank personnel.

The recall procedure involved two separate visits. During initial visits, the participants were informed about the process and asked to sign an informed consent form (including consent to the return of results). Their height, weight, blood pressure, pulse rate, and handgrip strength were recorded. Three blood samples collected from each participant were sent to Tartu University Hospital for biochemical analyses, and one sample was used for the secondary confirmation of the genetic findings with Sanger sequencing. The participants were asked about their family and personal medical histories to clarify the findings recorded in their EHRs and preceding events.

During the second recall visits, participants were informed of their genetic findings and counseled on the potential health ramifications, considering their medical histories, lifestyle choices, and biochemical analysis results. An experienced neurologist blinded to the participants’ genetic and medical backgrounds examined them and conducted TCS to detect LN + . The participants were counseled on their examination results and, when necessary, referred for further specialist visits. Detailed information on TCS LN+ and the medical devices used is provided in Supplementary Material S1. Brain MRI data were collected from participants’ EHRs and assessed by the neurologist.

### Analysis of p.His1069Gln variant carrier ancestry

Data from individuals with at least one copy of the p.His1069Gln variant, identified from NGS or imputed genotyping data (in 2023), were used to infer: (a) the p.His1069Gln frequency distribution across Estonian counties, (b) patterns of EstBB participant relatedness based on identity-by-descent (IBD) segment sharing, and (c) global ancestry profiles. Allele frequencies were reported by county of birth, disregarding self-reported ethnicity, or by self-reported ethnic group. Confidence intervals for allele frequencies were estimated using binom.test in R (v4.3.0) [27].

IBD segments were detected using IBIS (v1.20.8) [28] with the following calling parameters: -maxDist 0.1 -a 0.00138 -min_l 7 -mt 300 -er 0.004 -2 -min_l2 2 -mt2 150 -er2 0.008. As patterns of relatedness are affected by local demographics [29, 30], the pattern for p.His1069Gln carriers was compared with that for a random sample of EstBB participants matched according to sex, year and county of birth, and self-reported ethnicity. We estimated the average proportions of relatives (individuals sharing at least one IBD segment ≥7 cM) born in each county for the two focal sets, p.His1069Gln carriers and matched participants, and plotted the ratio of these two values on the Estonian map.

The ancestry of each EstBB participant was modeled as a mixture of global ancestry components, as described elsewhere [31]. We focused on Finnish- and Eastern-European–like ancestries, accounting for >90% of the modeled ancestry of 90% of EstBB participants. We grouped p.His1069Gln carriers (*n* = 2923) and non-carriers (*n* = 208,337) by the county of birth and divided the median carrier ancestry value by the non-carrier value for each county. Additional technical details are provided in Supplementary Material S1.

## Results

### Spectrum of *ATP7B* genetic variants among EstBB participants

**Fig. 2:**
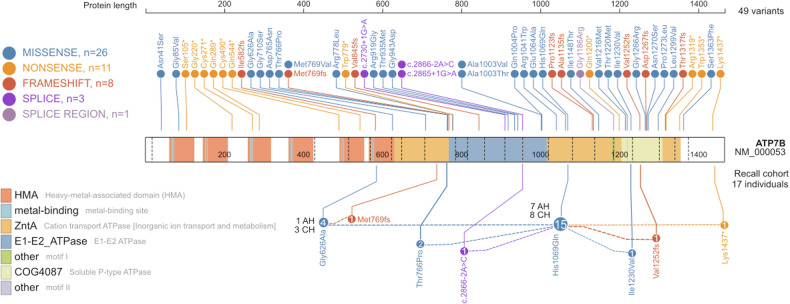
Pathogenic and likely pathogenic *ATP7B* variants detected in the EstBB cohort (above the protein) and the recall cohort (below the protein; *n* = 17). Numbers in circles indicate the numbers of variant carriers. Dashed lines connect compound heterozygote (CH) variants. AH alternative homozygote. This figure was originally created with the web application ProteinPaint (St. Jude Children’s Research Hospital, proteinpaint.stjude.org) and later modified to add relevant data.

Screening identified 1563 unique ATP7B variants. The first and second stages of filtration for pathogenicity yielded 121 and 106 variants, respectively. One deletion was added to the variant pool separately, as it was estimated to have a deleterious effect but did not conform to the algorithm criteria. Five of the 106 variants lacked sufficient data for ACMG classification but had potential for further analysis as they were specific to the Estonian population (Supplementary Table S1). The most frequent pathogenic *ATP7B* variant was c.3207 C > A (p.His1069Gln), with MAF 0.0059 (based on all carriers who overlapped in the genotyping array and imputed datasets (*n* = 2414)). Other variants were observed in fewer than five individuals in our NGS dataset. The final number of relevant P and LP variants was 49 (Fig. 2), of which three were yielded from additional exome sequencing (*n* = 4); all 49 variants were classified according to the 2015 ACMG/AMP guidelines (Supplementary Table S1).

### Genotype-based recall study results

Based on their genetic findings, 17 EstBB participants were selected for the recall study. One participant was deceased and another did not respond. The recall sample thus comprised 15 participants, resulting in a 93.7% positive response rate of all living recall participants. Nine participants were *ATP7B* compound heterozygotes and eight were alternative homozygotes (Fig. 2). The majority of the participants (15/17) were carriers of at least one copy of the p.His1069Gln allele and 7 were homozygous for this allele. Out of 17 participants, 8 had been diagnosed with WD before the recall. Seven (including four with previously undetected second pathogenic alleles) were prescribed WD treatment. The median age at diagnosis was 29 (range 9–53, SD 16) years.

The general metrics and objective data gathered during the recall visits were broadly within reference ranges (Table 1). Among the biochemical analyses performed, the serum ceruloplasmin and copper levels (WD-specific biochemical parameters) were below the reference thresholds for all recall participants (Table 2), and were significantly higher in homozygous carriers of the p.His1069Gln allele than in participants carrying no or one p.His1069Gln allele (Supplementary Fig. S2). Seven individuals out of 15 had alanine aminotransferase activity above the reference value and six had higher-than-normal gamma-glutamyl transferase activity. One individual showed elevated activity in all measured liver enzymes; this was likely compounded by a complex medical history, including cancer chemotherapy (goserelin).

**Table 1:** Summary of general metrics and objective data for the recall and EstBB cohorts.

	Recall participants (*n* = 15) mean ± SD	Biobank participants (*n* = 208,360) mean ± SD
Gender - female (%)	8 (53.3%)	136,378 (65.5%)
Age (range in yrs)	Median 43 ± 10 (31–66)	Median 48 ± 16 (18–104)
Height (cm)	171.9 ± 10.7	171.0 ± 9.4
Weight (kg)	81.8 ± 14.5	77.1 ± 17.3
BMI	28.0 ± 6.5	26.3 ± 5.3
		**Reference range**
Waist circumference (cm)	86.2 ± 12.6	M low risk <94 cm, high risk 94–102 cm, very high risk >102 cm; F low risk <80 cm, high risk 80–88 cm, very high risk >88 cm.**
Hip circumference (cm)	105.4 ± 9.3	
WHR	0.8 ± 0.1	M < 0.90, F < 0.85*
Systolic/diastolic (mm/Hg)	129 ± 15 / 81 ± 10	90/60–120/80**
Pulse (bpm)	67 ± 10	60–100**
Right hand (kg)	39 ± 11	
Left hand (kg)	35 ± 10	
Lefthandedness (%)	2 (13,3%)	

Normal reference values for WHR were provided by the World Health Organization [48] (marked with *).

Reference values for blood pressure, pulse, and waist circumferences were provided by the British Heart Foundation [49] (**).

Average blood pressure, pulse, and hand grip strength values were calculated from three repeated measures.

*BMI* body mass index, *M* male, *F* female, *WHR* waist–hip ratio.

**Table 2:** Biochemical findings from recall visit samples.

Biochemical analysis	Recall participants mean ± SD (*n* = 15)	Reference range
Leukocytes (E9/L)	7.3 ± 2.3	4.1–9.7
Erythrocytes (E12/L)	4.7 ± 0.5	F 4.1–5.2; M 4.5–5.7
Hemoglobin (g/L)	137.1 ± 8.1	F 121–150; M 134–170
Hematocrit (%)	41.3 ± 2.4	F 37–45; M 40–49
Mean corpuscular volume MCV (fL)	88.2 ± 5.2	82–95
Mean corpuscular hemoglobin MCH (pg)	29.2 ± 2.1	28–33
Mean corpuscular hemoglobin concentration MCHC (g/L)	331.6 ± 10.7	322–356
Red cell distribution width, coefficient of variation RDW-CV (%)	13.5 ± 1.7	12–15
Red cell distribution width, standard deviation RDW-SD (fL)	43.4 ± 4.87	38–48
Platelets (E9/L)	233.7 ± 83.5	157–372
Eosinophils (E9/L)(%)	0.1 (1.7%)	0.02–0.4
Basophils (E9/L) (%)	0.05 (0.6%)	0.01–0.08
Monocytes (E9/L) (%)	0.6 (8.5%)	0.24–0.8
Lymphocytes (E9/L) (%)	2.5 (35.9%)	1.3–3.1
Neutrophils (E9/L) (%)	4.0 (53.3%)	1.9–6.7
Immature granulocytes (E9/L)(%)	0.02 (0.3%)	0–0.03
Normoblasts NRBC (E9/L)(%)	0	0
High sensitivity CRP-hs (mg/L)	0.6 ± 0.6	Low CVD risk < 1.0; average risk 1.0–3.0; high risk > 3.0
Alanine aminotransferase ALAT (U/L)	47.3 ± 38.6	F < 35; M < 50
Aspartate aminotransferase ASAT (U/L)	35.0 ± 17.1	F < 35; M < 50
Alkaline phosphatase ALP (U/L)	86.3 ± 29.0	F 35–104; M 40–129
Gamma-glutamyl transferase GGT (U/L)	50.8 ± 33.7	F < 40; M < 60
Albumin (g/L)	44.3 ± 4.8	35–52
Lactate dehydrogenase LDH (U/L)	190.5 ± 46.5	< 250
Ceruloplasmin (g/L)	0.100 ± 0.023	F 0.16–0.45; M 0.15–0.30
Serum copper (mg/L)	0.4 ± 0.2	F 0.8–1.6; M 0.7–1.4

Reference values were provided by Tartu University Hospital United Laboratories, where the analyses were performed.

*CVD* cardiovascular disease, *CRP-hs* C-reactive protein (high sensitivity)

*F* female, *M* male, *SD* standard deviation.

### Neurological examination results

Thirteen (86.7%) of the 15 recall participants presented with neurological symptoms (Table 3). The most common symptom was postural tremor (66.7%), followed by dysarthria (53.3%) and hypersalivation (33.3%). MRI yielded no finding in three participants and revealed hyperintense lesions on T2/FLAIR sequences in nine (60.0%) participants; eight of these findings were considered to be non-specific to WD. TCS revealed LN+ in 11 (73.3%) individuals, of whom 7 had bilateral LN+ . Only one of the participants previously diagnosed with WD presented without LN+ .

**Table 3:** Main neurological findings in the recall cohort by symptom group.

Neurological trait	Count of affected individuals (out of 15)	Proportion (%)
**Tremors**	**10**	**66.7**
Action tremor	4	26.7
Postural tremor	10	66.7
Task-specific tremor - handwriting	2	13.3
Task-specific tremor - speaking	1	6.7
Rest tremor	1	6.7
**Oropharyngeal movement disorders**	**8**	**53.3**
Dysarthria	8	53.3
Dysphagia	4	26.7
Hypersalivation	5	33.3
**Ataxias**	**6**	**40**
Sensory ataxia	2	13.3
Static ataxia	3	20
Dynamic ataxia	4	26.7
**Other extrapyramidal movement disorders**	**5**	**33.3**
Dystonia	2	13.3
Rigidity	3	20
Bradykinesia	3	20
Choreathetosis	1	6.7
Spasticity	2	13.3
**Epilepsy**	0	0
**Earlier diagnosis of cognitive impairment**	0	0
**Mood disorders**	**3**	**20**
Depression	3	20
Mania	1	6.7
**Impairment of attention**	1	6.7
**MRI findings**		
MRI: midbrain atrophy	1	6.7
MRI: microangiopathic foci	5	33.3
MRI: hyperintense T2/FLAIR signals	9	60
MRI: no findings	3	20
**Transcranial sonography (TCS) findings**		
Hyperechogenicity of the lentiform nucleus (LN + )	11	73.3
LN+ bilaterality	7	46.7

### EstBB participants diagnosed with copper metabolism disorders

We found 59 EstBB participants who had been diagnosed with E83.0 at least once, according to EHR data. Apart from the 8 individuals enrolled in the recall study, no others had been formally diagnosed with WD despite having the E83.0 diagnosis code on record, the latter standing for “a copper metabolism disorder”, with no further specification. Twenty-five of these cases could not be classified due to the unavailability of epicrisis data (*n* = 1), enrollment in the recall study (*n* = 8), and insufficient data (*n* = 16). Among the remaining 34 individuals, neurological symptoms were most common (79.4%), followed by psychiatric symptoms (61.8%) and liver function abnormalities (58.8%; Table 4; Supplementary Fig. S3).

**Table 4:** Summary of main findings of the electronic health records analysis for EstBB participants diagnosed with copper metabolism disorders (*n* = 34).

	Participants with E83.0 (*n* = 34)
Gender - female (%)	19 (55.9%)
Age - median (range in yrs)	41.5 (24–85)
Neurological symptoms	27 (79.4%)
Psychiatric symptoms	21 (61.8%)
Hepatological symptoms	20 (58.8%)
Low ceruloplasmin (<0.15 g/L)	12 (35.3%)
Low serum copper (<0.7 mg/L)	8 (23.5%)
High 24 h copper excretion (>0.94 µmol/d)	5 (14.7%)
Genetic testing for WD in the healthcare system	10 (29.4%)
WD treatment	1 (2.9%)
Kayser-Fleischer rings	2 (5.8%)
**Categorization of WD likelihood**	
Unlikely WD	10 (29.4%)
Possible but unlikely WD	11 (32.4%)
Possible WD	13 (38.2%)

*WD* Wilson’s disease.

Twenty-eight (82.4%) of these individuals had overlapping symptoms related to the liver and nervous system (neurological or psychiatric). Twelve (35.3%) had low ceruloplasmin levels (of whom 7 individuals had no accompanying serum copper measurements) and 15 participants had no record of ceruloplasmin or copper measurement. Five individuals were reported to have high copper excretion in 24-h urine. Kayser-Fleischer rings had been described in two individuals. One participant had been prescribed WD treatment as a child, but the treatment had been discontinued due to side effects and poor adherence. We estimated that 29.4% of the 34 cases were likely undiagnosed WD, 38.2% were possibly WD, and 32.4% were possibly but unlikely to be WD. Six of the 13 possible WD candidates had been screened for pathogenic *ATP7B* variants, and single pathogenic variants were detected in two cases.

### Copper measurement data at the EstBB

**Fig. 3:**
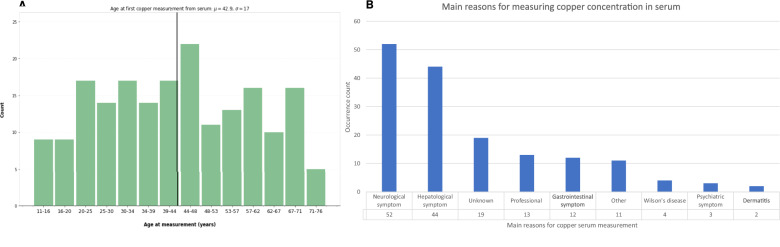
Serum copper measurement data at the Estonian Biobank. **A** Distribution of EstBB participants’ age at first serum copper measurement, based on EHR data. The solid black line denotes the median. **B** Main reasons for serum copper measurement, according to EHRs.

Laboratory serum copper measurement data from EHRs were used to investigate the indications for copper testing in Estonia in order to identify potential diagnostic bottlenecks. The indications were analysed using EstBB epicrises, ICD-10 codes, and age at first measurement. Initially, 355 serum and 24-h urine copper measurements were retrieved. After the removal of duplicates (*n* = 91) and measurements with unclear units (*n* = 4), 190 serum copper measurements from 164 individuals and 49 24-h urine copper measurements from 47 individuals remained. The median age at first copper measurement was 42.9 years (Fig. 3A). The most common reasons for referral for testing were neurological symptoms (*n* = 52) and liver disease (*n* = 44; Fig. 3B). Other reasons included routine professional checks, gastrointestinal symptoms, and psychiatric symptoms. For 19 (11.6%) individuals, the data were insufficient to determine the indication for serum copper measurement.

### Clinical prevalence of WD and copper metabolism disorders in Estonia

According to the Estonian Health Insurance Fund [32], the code E83.0 had been recorded for 146 current patients in Estonia on October 12, 2023, and this number had increased annually by 1–7 individuals over the last 10 years. These data suggest that copper metabolism disorders affect about 1 in 9400 Estonians. To more specifically estimate the number of individuals diagnosed with WD, we used data from the Estonian Agency of Medicine’s 2023 statistical yearbook [33]. The number of defined daily doses sold for WD medications [D-penicillamine and zinc-based pharmaceuticals (i.e. zinc acetate)] was approximately 0.01/1000 inhabitants/day for either, suggesting that about 14 people in Estonia use either medication regularly.

### Ancestry of p.His1069Gln variant carriers

**Fig. 4:**
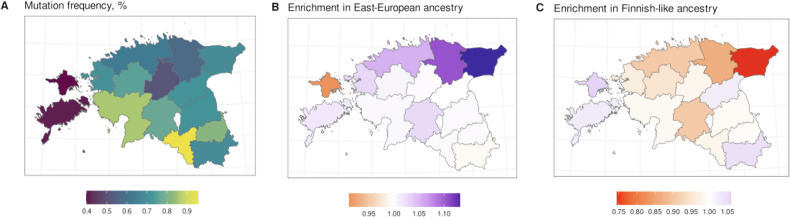
Distribution of p.His1069Gln carriers by county of birth in Estonia. **A** p.His1069Gln allele frequencies (%) among EstBB participants by county of birth in Estonia. Enrichment in Eastern European–like **B** and Finnish-like **C** ancestry in p.His1069Gln carriers relative to non-carriers by county of birth.

We identified 2923 individuals with at least one p.His1069Gln allele from NGS or imputed genotype data. Allele frequency was estimated for all EstBB participants with available county of birth information (*n* = 198,092, of whom 2722 carried one copy of p.His1069Gln); (Fig. 4A, Supplementary Table S2).

For downstream comparisons, a control set of non-carriers matching p.His1069Gln carriers in self-reported ethnicity, sex, year, and county of birth was used. Matches were unavailable for 19 carriers, resulting in 2904 participants in both carrier and matched non-carrier sets. We identified the proportion of each query dataset (carriers, matched non-carriers, and remaining 205,464 EstBB participants) to which each p.His1069Gln carrier and matched non-carrier were related (Supplementary Fig. S4; see Materials and Methods for relatedness definition). The distributions of carriers’ and matched non-carriers’ relatedness were very similar, but the variant carriers’ query set contained more relatives than the matched sample did.

We examined the proportions of relatives in each county compared to the distribution for matched non-carriers (Supplementary Fig. S5). The distributions were similar, but p.His1069Gln carriers showed a slight increase in southern Estonian and a decrease in northern Estonian ancestry, aligning with the variant’s frequency distribution. Carriers born in southern Estonia showed little difference from non-carriers, while those from northeastern Estonia and Hiiumaa Island had less typical global ancestry profiles, as reflected in the median ancestry ratios. (Fig. 4B, C).

The global frequency of the most prevalent pathogenic *ATP7B* variant (p.His1069Gln, rs76151636) is 0.0014 according to dbSNP Build 156 [34], whereas in direct and imputed EstBB data it is 0.0059 - almost five times higher. Based on this estimate, we extrapolated that the Estonian population includes 47 (1:29,000) p.His1069Gln alternative homozygous individuals. Considering the proportion of compound heterozygotes in our recall study, we estimated that the Estonian population includes about 60 (1:22,760) compound heterozygotes. In the FinnGen population, the p.His1069Gln allele frequency is 0.0008 [35], more than seven times lower than the estimate for the EstBB population. Based on data from FinnGen’s open database Risteys (v2.2.0) [36], the proportion of individuals diagnosed with copper metabolism disorders (ICD-10 code E83.0) was also significantly larger in the EstBB than in the FinnGen population (0.028% vs. 0.0078%; *p* < 0.001, Fisher’s exact test [37]).

## Discussion

Here, we demonstrate the potential of combined genetic and phenotypic data from a population-based biobank in a multilayer analysis of WD in Estonia and the power of a genotype-based recall study in detecting underdiagnosed WD cases. The majority of our recall study participants could be classified into two categories: those with pronounced symptom onset requiring immediate healthcare attention early in life, and those with few reported WD-like symptoms into their 30s and beyond. However, the seemingly healthy condition of the latter group often masked underlying issues, as indicated by ceruloplasmin and copper levels, neurological findings, and, in some cases, liver enzyme activity. These findings indicate that all participants in our recall cohort showed signs of WD that, if left untreated, could lead to health issues of varying severity in the future.

All recall study participants had low ceruloplasmin and serum copper levels and the majority had neurological symptoms, irrespective of genotype, prior WD diagnosis, and age. MRI results were less specific to WD than TCS results, possibly explained by LN+ being a highly specific marker for copper accumulation [38]. The percentage of recall study participants with LN+ is comparable to those found in other studies [12, 13]. Although a wide age range at the time of WD diagnosis is not uncommon [39], diagnosis at later ages has been linked to larger proportion of p.His1069Gln carriers [40]. Our findings may also reflect this trend.

Only a few nationwide WD epidemiological studies have been conducted using national registries [41–43]. To our knowledge, this report is the first to describe a large-scale national biobank based study. Such an approach avoids the potential bias of patient-derived cohort analyses and may partly explain the high observed frequency of p.His1069Gln relative to those in other populations. In a study conducted in Finland and involving the analysis of EHRs and registry data covering 1998–2017, 33 individuals were determined to have WD and all were confirmed to have received treatment for it [41]. This number is comparable to the number of individuals in Estonia who received WD treatment in 2023 alone, although the population of Finland is approximately four times larger than that of Estonia. The rarity of WD (and p.His1069Gln) in Finland has been documented previously [5, 40]. This observation supports the theory that p.His1069Gln arose in Eastern Europe [4].

Generally, the population of northeastern Estonia has been shown to have greater genetic similarity to Finns and less to Latvians and Lithuanians relative to the Estonian average [30, 44]. This observation supports our finding that p.His1069Gln carriers in Estonia have a more southern Estonian–like genetic profile (and thus a larger Eastern-European ancestry component); the Finnish population also has a lower frequency of this variant, as seen with FinnGen.

The high frequency of p.His1069Gln seen in EstBB correlates with the high prevalence of E83.0 diagnoses in Estonian national statistics. The significant gap between the number of patients with WD receiving treatment and the number of individuals diagnosed with copper metabolism disorders in Estonia warrants further research. Some patients with formal WD diagnoses may not require current treatment (as we have seen in our recall cohort), but this group is likely to be small or to not account for all untreated individuals. Other very rare copper metabolism disorders, such as Menkes disease and occipital horn syndrome, have been described [45]; however, the EstBB participants’ EHRs contained no reference to these diseases. Considering the prevalence of p.His1069Gln in the EstBB population, we find it more likely that a considerable number of individuals remain undiagnosed with WD or misdiagnosed. Genetic screening holds promise for the identification of undiagnosed WD cases and potential prevention of health complications, as demonstrated elsewhere [46]. Given the polymorphic nature of *ATP7B*, we would ideally recommend a hypothesis-free sequencing method for this. About 40% of all individuals diagnosed with copper metabolism disorders in Estonia are EstBB participants, further emphasizing the potential value of biobanks in the study of rare diseases. The large percentage (>90%) of biobank participants who responded positively to the recall study invitations reflects continued interest in biobank activities and personalized genetic feedback.

Most serum copper measurements examined in this study were ordered due to the presence of neurological and liver-related symptoms suspicious for copper metabolism disorder, as expected. Our analysis showed that psychiatric symptoms alone were generally not considered to be an indication for serum copper testing. Given the growing evidence that psychiatric symptoms develop before neurological and liver-related manifestations in WD, causing diagnostic delay [47], it may be prudent to consider WD in otherwise unexplainable cases of psychiatric issues in young individuals. Additionally, we recommend the consideration of copper testing for all individuals presenting with neurological/psychiatric and liver-related symptoms at a young age, and of Eastern European origin as a possible supporting criterion when WD-like symptoms are present.

As the EHRs analyzed in this study dated back about 20 years, earlier blood copper concentration data may exist for some of the included individuals. We consider this to be unlikely, as the EHR data showed that physicians reordered copper or ceruloplasmin measurements on rare occasions after excluding WD as a diagnosis. In our experience, repeat copper measurement would most likely occur in the case of an established WD diagnosis or for professional reasons.

In conclusion, our findings show a continuing need for the implementation of personalized medicine approaches and population-based genetic screening for rare disorders with multifaceted clinical presentations. When such measures are available and applied in the healthcare system, timely diagnosis and appropriate treatment can significantly improve patient outcomes. The multidisciplinary management of individuals with copper metabolism disorders is an important aspect of follow-up and treatment due to the diverse symptom spectra of these conditions. Our analysis showed that p.His1069Gln, the most frequent pathogenic *ATP7B* variant in Estonia, is associated more with an Eastern-European ancestry component and has a notably higher frequency in Estonia than in any known report to date. Based on the results from both genotypic and phenotypic datasets from a population based national biobank, we consider WD to be underdiagnosed and undertreated in Estonia.

## Supplementary information

Supplementary Table S2

Supplementary Table S1

Supplementary Material S1

Supplementary Figures

## Data Availability

The genotypic and phenotypic data from the Estonian Biobank are available under restricted access and can be obtained with the permission of the Estonian Committee on Bioethics and Human Research. Data pertaining to *ATP7B* variants present in the recall cohort have been submitted to the ClinVar database under accession numbers SCV004239217–SCV004239222.
