# Supplementary material for: Use of Estonian Biobank data and participant recall to improve Wilson’s disease management
Source: Eur J Hum Genet. 2024 Dec 14;33(11):1499–508. doi: 10.1038/s41431-024-01767-9 (PMC12583600; doi:10.1038/s41431-024-01767-9)
Supplement: Supplementary file 1 — Supplementary Table S2 [file 41431_2024_1767_MOESM1_ESM.pdf]

E	F	G	H	I	J	K	L	M	N	O	P
Non-carriers, Finnish-like, %*	Fraction of relatives in non-carriers set	Fraction of relatives in carrier set	Number of potential relatives	Carriers with a match	Average number of relatives per 1 non-carrier (wild-type homozygotes)	Average number of relatives per 1 carrier (heterozygotes + alternative homozygotes)	Allele frequency (%)	Total	Wild-type homozygotes (p.H1069Q)	Alternative homozygotes (p.H1069Q)	Heterozygotes (p.H1069Q)
28.7	8.5820104	8.535715	51841	670	4449	4425	0.63	53181	52507	2	671
30.8	9.8534613	9.4997473	1979	16	195	188	0.4	2011	1995	0	16
29.5	6.0451505	5.9113712	11960	169	723	707	0.69	12298	12126	0	170
31.3	9.7284093	9.6996695	6959	72	677	675	0.51	7103	7029	0	73
30.9	10.067692	10.116923	8125	119	818	822	0.72	8363	8243	0	120
26.6	9.2445328	9.1699801	4024	59	372	369	0.71	4142	4083	0	59
39.8	8.4568439	8.2970067	13764	162	1164	1142	0.57	14088	13926	0	162
23.6	10.790872	11.04095	15995	283	1726	1766	0.86	16561	16276	0	284
16.8	13.541134	13.915858	5871	94	795	817	0.82	6059	5960	0	99
29	9.6343693	9.6709324	5470	84	527	529	0.76	5638	5552	1	84
26.4	8.6849019	8.6076341	6471	53	562	557	0.41	6577	6523	0	54
22.5	11.106524	11.271676	31486	459	3497	3549	0.72	32404	31937	3	458
17.2	12.059701	12.283582	6700	131	808	823	0.96	6962	6830	1	131
23	11.54668	11.727482	12168	194	1405	1427	0.77	12556	12362	0	194
14	14.375443	14.507142	9871	139	1419	1432	0.69	10149	10009	0	140
8.4	3.8106416	3.7871674	12780	200	487	484	0.76	13180	12979	0	201

A	B	C	D
County	Carriers, EasternEurope-like, %*	Non-carriers, EasternEurope-like, %*	Carriers, Finnish-like, %*
Harju	71.1	67.5	26
Hiiu	48.9	53.7	32.6
Ida-Viru	76.4	67.5	21.95
Järva	66	65.9	30.7
Jõgeva	67.8	67.8	31.6
Lääne	69.2	67.3	25.6
Lääne-Viru	63.2	57.2	34.5
Pärnu	75.1	74.8	23.3
Põlva	83.2	83	16.7
Rapla	67.9	67.5	27.5
Saare	64.95	63.9	27.1
Tartu	77	76.8	22.3
Valga	82.7	82.5	16.85
Viljandi	78	76.1	20.9
Võru	85.4	85.8	14.6
no info	89.3	85.5	5

Maps	columns used
Figure 3A	F
Figure 3B	ratio G/H
Figure 3C	ratio M/N
Supplementary Figure S5	ratio O/P

*Median ancestry value
