# Supplementary material for: Use of Estonian Biobank data and participant recall to improve Wilson’s disease management
Source: Eur J Hum Genet. 2024 Dec 14;33(11):1499–508. doi: 10.1038/s41431-024-01767-9 (PMC12583600; doi:10.1038/s41431-024-01767-9)
Supplement: Supplementary file 2 — Supplementary Table S1 [file 41431_2024_1767_MOESM2_ESM.pdf]

Position (RefSeq build GRCh37)	SNV ID	Nucleotide change*	Amino acid change	Consequence	WilsonGen assessment	ClinVar assessme nt	EstBB assessm ent	ACMG/AMP 2015 classification codes
13:52508981	rs768833241	c.4309A>T	p.Lys1437Ter	stop-gained	-	-	P	PVS1 PM2 PM4 PP3
13:52509765	rs776848753	c.4088C>T	p.Ser1363Phe	missense	VUS	LP/P	P	PS1 PM2 PM3 PP3 PP4 PP5
!3:52509795	rs193922110	c.4058G>A	p.Trp1353Ter	stop-gained	P	LP/P	P	PVS1 PS1 PM2 PM4 PP3 PP5
13:52511478	rs193922109	c.3955C>T	p.Arg1319Ter	stop-gained	P	P	P	PVS1 PS1 PM1 PM4 PP3 PP5
13:52511484	rs1057516228	c.3948del	p.Thr1317LeufsTer13	frameshift	P	LP	P	PVS1 PM1 PM2 PM4 PP3
13:52511620	rs749472361	c.3895C>G	p.Leu1299Val	missense	-	-	LP	PM1 PM2 PM5 PP3
13:52511697	rs758355520	c.3818C>T	p.Pro1273Leu	missense	P	P	LP	PS1 PM1 PP3 PP6
13:52511706	rs121907990	c.3809A>G	p.Asn1270Ser	missense	P	P	LP	PS1 PM1 PM3 PP3
13:52511714	rs1057516740	c.3800del	p.Asp1267ValfsTer63	frameshift	P	LP	P	PVS1 PM1 PM2 PM4 PP3
13:52511719	rs121907992	c.3796G>A	p.Gly1266Arg	missense	P	P	P	PS1 PS3 PM1 PM2 PM3 PP3 PP4 PP5
13:52513198	rs200911496	c.3688A>G	p.Ile1230Val	missense	LP	CI	LP	PM2 PM3 PP3 PP4
13:52513227	rs193922107	c.3659C>T	p.Thr1220Met	missense	LP	LP/P	P	PS1 PM1 PM2 PM3 PP3 PP5
13:52513240	rs776280797	c.3646G>A	p.Val1216Met	missense	LP	LP/P	LP	PM1 PM3 PP3 PP4 PP5
13:52513288	rs786204658	c.3598C>T	p.Gln1200Ter	stop-gained	P	LP	P	PVS1 PM1 PM2 PP3
13:52515217	rs786204547	c.3556G>C	p.Gly1186Arg	missense	LP	LP/P	LP	PM1 PM2 PM3 PP3 PP4 PP5
13:52515330	rs60431989	c.3443T>C	p.Ile1148Thr	missense	LP	P	P	PS1 PM1 PM2 PP3 PP5
13:52516531	rs137853281	c.3402del	p.Ala1135GlnfsTer13	frameshift	P	P	P	PVS1 PS1 PM1 PM2 PM4 PP3 PP4 PP5
13:52516540	rs1312634432	c.3368_3393del	p.Pro1123GlnfsTer21	frameshift	-	-	P	PVS1 PM2 PP3
13:52518281	rs76151636	c.3207C>A	p.His1069Gln	missense	P	P	P	PS1 PS3 PS4 PM1 PP1 PP3 PP4 PP5
13:52518297	rs374094065	c.3191A>C	p.Glu1064Ala	missense	LP	LP/P	P	PS1 PM1 PM3 PM5 PP1 PP3 PP4 PP5
13:52518367	rs746485916	c.3121C>T	p.Arg1041Trp	missense	LP	LP/P	P	PS1 PM1 PM2 PM3 PP3 PP4 PP5
13:52520469	rs587783307	c.3011A>C	p.Gln1004Pro	missense	LP	P	P	PS1 PM1 PM2 PP3 PP5

13:52520472	rs775055397	c.3008C>T	p.Ala1003Val	missense	LP	LP/P	LP	PS1 PM1 PP3 PP4 PP5
13:52520473	rs201497300	c.3007G>A	p.Ala1003Thr	missense	P	LP/P	P	PS1 PM1 PM2 PM3 PP1 PP3 PP4 PP5
13:52523797	rs587783306	c.2865+1G>A	-	splice donor site	P	LP/P	P	PVS1 PM2 PP3
13:52523835	rs779323689	c.2828G>A	p.Gly943Asp	missense	LP	LP/P	P	PS1 PM1 PM2 PP3 PP4 PP5
13:52523859	rs750019452	c.2804C>T	p.Thr935Met	missense	LP	LP/P	P	PS1 PS4 PM1 PM2 PM3 PP3 PP4 PP5
13:52523908	rs121907993	c.2755C>G	p.Arg919Gly	missense	LP/P	P	P	PS1 PM1 PM2 PM3 PP3 PP4 PP5
13:52524142	rs1057516425	c.2730+1G>A	-	splice donor site	P	LP	P	PVS1 PM2 PP3
13:52524450	rs755709270	c.2532delA	p.Val845SerfsTer28	frameshift	P	P	P	PVS1 PS1 PM1 PM2 PM3 PM4 PP3 PP4 PP5
13:52532466	rs137853283	c.2336G>A	p.Trp779Ter	stop-gained	P	P	P	PVS1 PS1 PM1 PM2 PP3 PP4 PP5
13:52532469	rs28942074	c.2333G>T	p.Arg778Leu	missense	P	P	P	PS1 PM1 PM2 PM3 PP3 PP4 PP5
13:52532497	rs137853287	c.2304dupC	p.Met769HisfsTer26	frameshift	P	P	P	PVS1 PS1 PM1 PM4 PP1 PP3 PP4 PP5
13:52532497	rs193922103	c.2305A>G	p.Met769Val	missense	VUS	LP/P	P	PS3 PM1 PM2 PM3 PP3 PP4 PP5
13:52532509	rs28942075	c.2293G>A	p.Asp765Asn	missense	VUS	P	P	PS1 PM1 PM2 PP3 PP5
13:52532674	rs137853285	c.2128G>A	p.Gly710Ser	missense	LP	P	P	PS1 PM1 PM2 PM3 PP3 PP4 PP5
13:52536042	rs587783299	c.1877G>C	p.Gly626Ala	missense	LP	CI	LP	PM1 PM2 PM3 PP3 PP4 PP5
13:52539130	rs753962912	c.1745_1746delTA	p.Ile582ArgfsTer25	frameshift	P	LP/P	P	PVS1 PM1 PM2 PM4 PP3 PP4 PP5
13:52542657	rs766906034	c.1630C>T	p.Gln544Ter	stop-gained	P	-	P	PVS1 PM1 PM2 PP3
13:52544701	rs778675259	c.1470C>A	p.Cys490Ter	stop-gained	P	LP/P	P	PVS1 PM1 PM2 PP3 PP5
13:52548491	rs121907999	c.865C>T	p.Gln289Ter	stop-gained	P	LP/P	P	PVS1 PM2 PP3 PP4 PP5
13:52548543	rs572147914	c.813C>A	p.Cys271Ter	stop-gained	P	P	P	PVS1 PS1 PP3 PP4 PP5
13:52548698	rs1229196914	c.658G>T	p.Gly220Ter	stop-gained	-	-	P	PVS1 PM2 PP3

13:52549042	rs753236073	c.314C>A	p.Ser105Ter	stop-gained	P	P	P	PVS1 PM1 PM2 PM3 PP3 PP5
13:52549102	rs786204643	c.254G>T	p.Gly85Val	missense	P	LP	LP	PS3 PM1 PM2 PP3
13:52549234	rs201738967	c.122A>G	p.Asn41Ser	missense	LP	LP/P	P	PS1 PS3 PM3 PP3 PP4 PP5
13:52511760	novel	c.3754del	p.Val1252SerfsTer78	frameshift	-	-	P	PVS1 PM2 PM3 PM6 PP3 PP4
13:52532506	CM1210109	c.2296A>C	p.Thr766Pro	missense	-	-	LP	PM2 PM3 PM5 PP1 PP3 PP4
13:52520616	rs1377418826	c.2866-2A>C	-	splice acceptor site	-	P	P	PVS1 PM2 PM3 PM6 PP3 PP4

*All variants have been mapped to *ATP7B* transcript NM_000053.4.
VUS - variant of uncertain significance, LP - likely pathogenic, P - pathogenic, CI - conflicting information.

Position (RefSeq build GRCh37)	SNP ID	Nucleotide change*	Amino acid change	Consequence	Overlap with regulatory element (Ensembl ID)	Allele count in EstBB NGS dataset	CADD score	WilsonGen assessment	ClinVar assessment	EstBB assessment (population frequency comparison with GnomAD)
13:52513252	novel	c.3634A>G	p.Met1212Val	missense variant	-	1	24,7	-	-	research potential, novel variant
13:52517050	rs1216657292	c.3244-360C>T	-	intron variant	Promoter flanking region (ENSR00000978792)	4	15,1	-	-	research potential, specific to Estonian population
13:52523926	rs1209293534	c.2737A>G	p.Ile913Val	missense variant	-	3	23,3	-	-	research potential, specific to Estonian population
13:52532506	CM1210109	c.2296A>C	p.Thr766Pro	missense variant	-	1	27,5	-	-	research potential, specific to Estonian population? No gnomAD data
13:52516680	novel	c.3254C>A	p.Thr1085Lys	missense variant	Promoter flanking region (ENSR00000978792)	1	24,7	-	-	research potential, specific to Estonian population? No gnomAD data

*All variants have been mapped to *ATP7B* transcript NM_000053.4.
