# Supplementary material for: Use of Estonian Biobank data and participant recall to improve Wilson’s disease management
Source: Eur J Hum Genet. 2024 Dec 14;33(11):1499–508. doi: 10.1038/s41431-024-01767-9 (PMC12583600; doi:10.1038/s41431-024-01767-9)
Supplement: Supplementary file 3 — Supplementary Material S1 [file 41431_2024_1767_MOESM3_ESM.docx]

**Supplementary Material S1**

**List of abbreviations**

ACMG – American College of Medical Genetics

AMP – American Molecular Pathologists

CVD – cardiovascular disease

CRP-hs – C-reactive protein, high sensitivity

EstBB – Estonian Biobank

EHRs – Electronic health records

F – female

M – male

IBD - identity-by-descent

ICD-10 – International Classification of Disease, 10th edition

LP – likely pathogenic

MAF – Minor allele frequency

MRI – Magnet resonance imaging

NGS – Next-generation sequencing

P – pathogenic

SD – standard deviation

TCS – Transcranial Sonography

WD – Wilson’s disease

WES – Whole exome sequencing

WGS – Whole genome sequencing

WHR – waist-to-hip ratio

**Follow-up exome sequencing**

DNA was extracted from venous blood collected in K2EDTA blood collection tubes. DNA was extracted using QIAGEN QIAmp DNA Blood Mini kit reagents. The exome sequencing was conducted as follows in the Institute of Genomics Core Facility, University of Tartu, Estonia. Extracted DNA samples were quantified with Qubit and exome libraries were prepared using Illumina DNA Prep with Exome 2.5 Enrichment kit. The libraries were quantified with Qubit, library profiles were assessed with TapeStation 4200 System. Sequencing was carried out on an Illumina NextSeq2000 using NextSeq 2000 P3 Reagents (200 Cycles) in the paired-end 2 x 101 bp mode. Post-sequencing analysis was performed with onboard DRAGEN Enrichment App v 3.10.12, using the HG38 BED file provided by the library preparation kit manufacturer^1^ and Homo sapiens [1000 Genomes] hg38 Custom Alt Aware Graph reference genome^2^.

^1^https://support.illumina.com/downloads/Illumina-dna-prep-exome-20-bed-
files.html
^2^Source: http://ftp.1000genomes.ebi.ac.uk/vol1/ftp/technical/reference/
GRCh38_reference_genome/GRCh38_full_analysis_set_plus_decoy_hla.fa
with an Illumina modified ALT-to-GRCh38 alignment file, originating from
http://sourceforge.net/projects/bio-bwa/files/bwakit/bwakit-0.7.12_x64-linux.tar.bz2/download, augmented with proprietary population alt haplotypes

**Transcranial Sonography**

An experienced neurologist performed transcranial sonography (TCS) during the 2^nd^ recall visit on all available recall participants. The TCS was conducted through the preauricular acoustic bone window using a 2.5-MHz S4-2 FAST transducer in two-dimensional mode (Mindray ultrasound system, a color portable MX7 ultrasound system with the ZST+ Platform; ZONARE Medical Systems Inc., USA). Following the identification of the mesencephalic brainstem, attention was focused on the ipsilateral LN region, where hyperechogenic areas visualized on the monitor were and the image was captured. LN echogenicity was assessed independently from the right and left temporal windows.

**Other medical devices used for recall visits**

Dynamometer - Patterson Medical Jamar Plus+ Hydraulic Hand Dynamometer

Blood tubes – A) 3 tubes used for biochemical analyses – K2E/K3E EDTA tube, lithium-heparin tube, microelements tube with clotting activator, B) K2E/K3E EDTA tube for secondary validation with Sanger sequencing, C) PAXGene blood RNA tube (BD Biosciences)

**IBD segment analysis**

We used ggplot2 v3.4.2 (Wickham 2016) and sf v1.0.13 (Pebesma 2018, Pebesma & Bivand, 2023) packages in R v4.3.0 for creating maps. Shp objects used to plot maps of Estonia with county borders were retrieved from the Estonian Land Board website (administrative and settlement units, 2023.02.01, <https://geoportaal.maaamet.ee/eng/Spatial-Data/Administrative-and-Settlement-Division-p312.html>).

R Core Team (2023). _R: A Language and Environment for Statistical Computing_. R Foundation for Statistical Computing, Vienna, Austria. <<https://www.R-project.org/>>.

H. Wickham. ggplot2: Elegant Graphics for Data Analysis. Springer-Verlag New York, 2016.

Pebesma, E., 2018. Simple Features for R: Standardized Support for Spatial Vector Data. The R Journal 10 (1), 439-446, https://doi.org/10.32614/RJ-2018-009

Pebesma, E., & Bivand, R. (2023). Spatial Data Science: With Applications in R (1st ed.). Chapman and Hall/CRC. https://doi.org/10.1201/9780429459016
