# Supplementary material for: Use of Estonian Biobank data and participant recall to improve Wilson’s disease management
Source: Eur J Hum Genet. 2024 Dec 14;33(11):1499–508. doi: 10.1038/s41431-024-01767-9 (PMC12583600; doi:10.1038/s41431-024-01767-9)
Supplement: Supplementary file 4 — Supplementary Figures [file 41431_2024_1767_MOESM4_ESM.pdf]

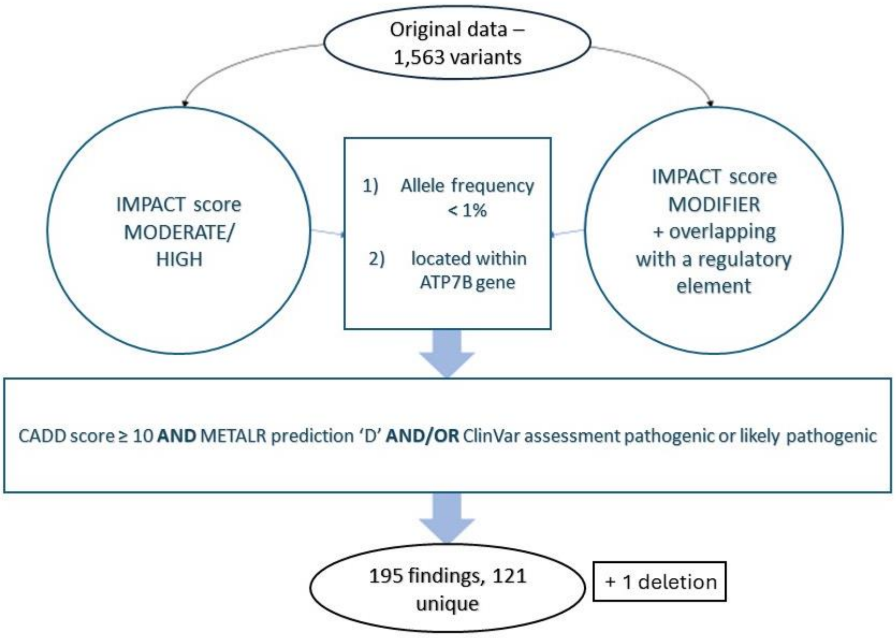

Supplementary Fig. S1. First stage of *ATP7B* variant filtration.

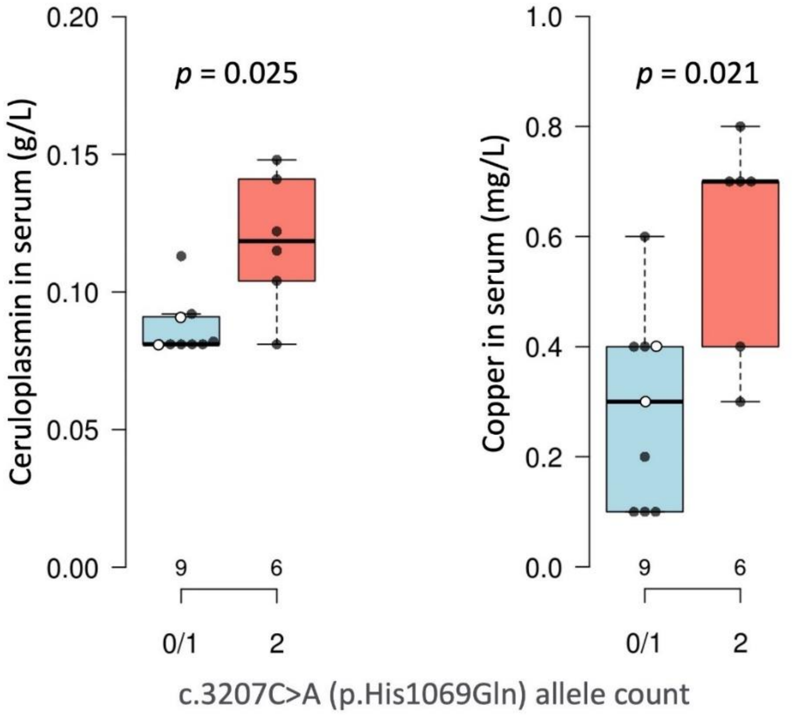

Supplementary Fig. S2. Serum ceruloplasmin and copper levels in the recall cohort. White dots represent p.His1069Gln variant non-carriers. Numbers of individuals are shown under the boxplots. *P* values were calculated using the Mann–Whitney *U* test. Thick lines indicate medians and whiskers denote 1.5× interquartile ranges from the first to the third quartiles.

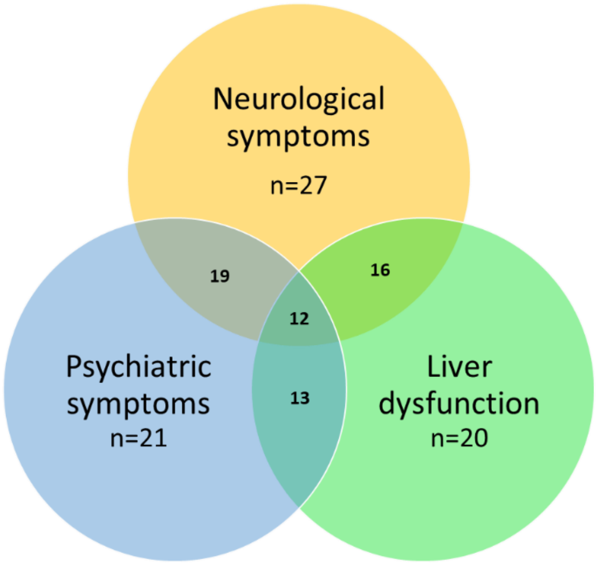

Supplementary Fig. S3. Symptom sets among EstBB participants diagnosed with copper metabolism disorders (*n* = 34).

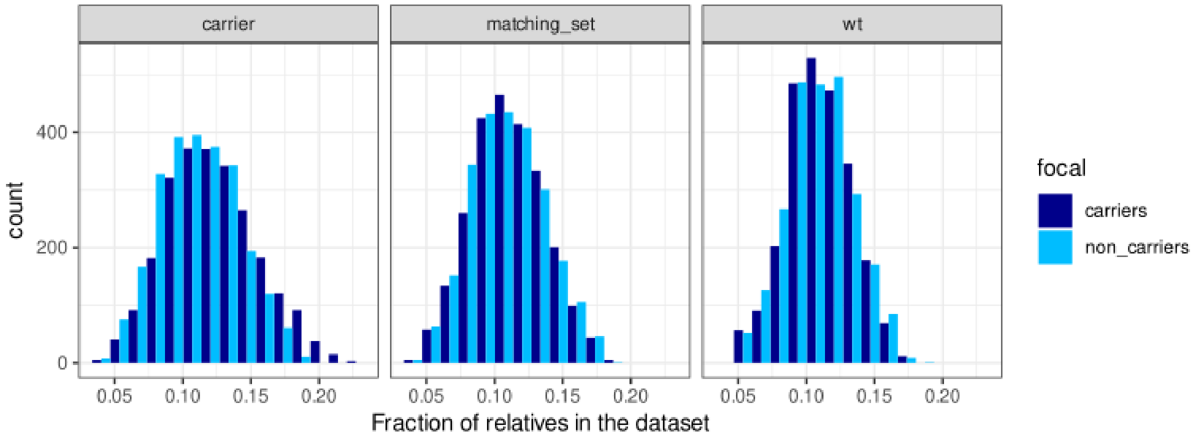

Supplementary Fig. S4. Proportions of relatives of p.His1069Gln carriers and matched non-carriers among p.His1069Gln carriers, matched non-carriers, and remaining non-carriers.

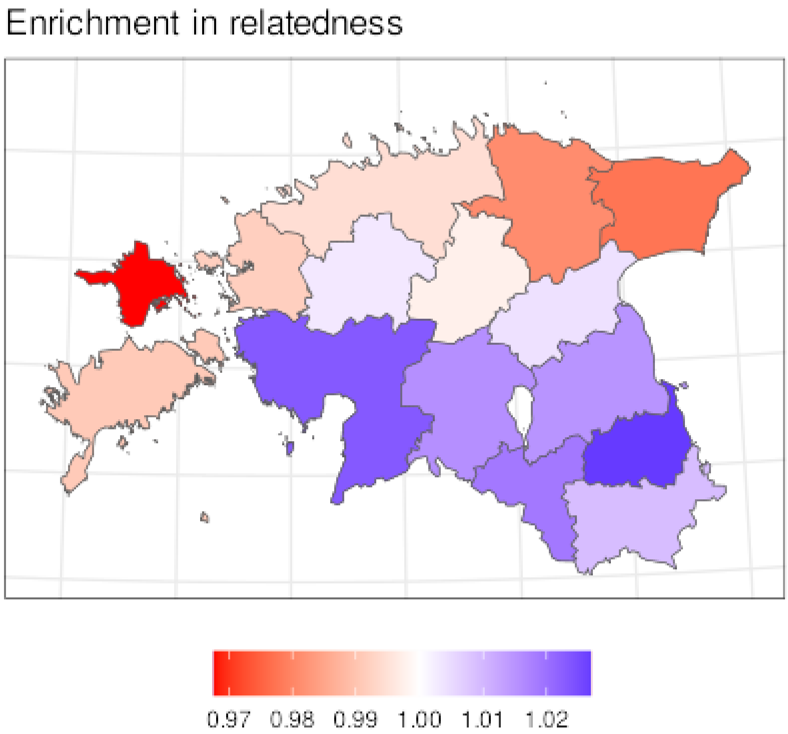

Supplementary Fig. S5. Patterns of relatedness for EstBB participants carrying the p.His1069Gln variant, mapped by county of birth. Dark-blue and red values indicate more and fewer average numbers of relatives born in the given counties relative to the relatedness of matched non-carriers, respectively.
